# Body roundness index and adolescent metabolic syndrome: dose–response analysis and serum biomarker mediation

**DOI:** 10.3389/fnut.2026.1930822

**Published:** 2026-09-16

**Authors:** Hui Yao, Yiwen Wang, Fang Zhou, Fei Wang, Xiaochen Wang, Runyu Du

**Affiliations:** 1 Department of Endocrinology, Shengjing Hospital of China Medical University, Shenyang, China; 2 Department of Pediatrics, Shengjing Hospital of China Medical University, Shenyang, China; 3 Department of Endocrinology, Liaoning Provincial People’s Hospital, Shenyang, China

**Keywords:** adipokine, adolescent, body roundness index, dose–response analysis, mediation analysis, metabolic syndrome, restricted cubic spline

## Abstract

**Background:**

The Body Roundness Index (BRI) combines waist circumference and height into a single geometric measure that is intended to reflect visceral adiposity. Its ability to predict metabolic syndrome (MetS) in adolescents and the biological processes that mediate this association have not been well defined. We tested the BRI-MetS relationship in Chinese adolescents, compared BRI with body mass index (BMI), and examined whether circulating biomarkers mediated this association.

**Methods:**

This cross-sectional study recruited 914 Chinese adolescents (53.2% boys) aged 11–16 years. MetS was defined according to Chinese pediatric guidelines (CN2025) and the International Diabetes Federation (IDF) criteria. Associations and predictive performance were assessed using logistic regression, restricted cubic splines (RCS), and receiver operating characteristic (ROC) curves. Subgroup analyses were stratified by sex and BMI category. Potential mechanistic pathways involving 22 serum biomarkers were investigated by correlation networks, hierarchical clustering, and two-stage mediation models.

**Results:**

BRI was positively associated with MetS (CN2025: OR = 3.63 per unit, 95%CI 2.91–4.53; IDF: OR = 2.64, 95%CI 2.14–3.26). RCS demonstrated non-linear dose–response relationship, with an acceleration in risk beyond BRI of approximately 4.0. BRI outperformed BMI in discriminating MetS (AUC 0.873 vs. 0.835, *p* = 0.005), especially among normal-weight participants (OR = 3.48, *p* < 0.001). Sex stratification showed that BRI was associated with hypertriglyceridemia and hypertension in male adolescents (*p* < 0.001) but not in female adolescents. We identified three biomarker co-expression modules: the upward module (ALT, FINS, and UA) mediated 52.9% of the association between BRI and hypertriglyceridemia and 54.1–59.5% of the association between BRI and hypertension; SFRP5 and irisin mediated the associations of BRI with fasting plasma glucose (FPG) and HDL-cholesterol, respectively.

**Conclusion:**

BRI may be a better predictor of MetS in Chinese adolescents, particularly when MetS is defined using domestic criteria. Its predictive power was greatest among normal-weight adolescents and varied by sex. Exploratory mediation analyses identified the metabolic-inflammatory axis and protective adipokines as key mediators, suggesting hypothesis-generating targets for interventions to improve adolescent metabolic health that require longitudinal validation.

## 1 Introduction

The increasing prevalence of childhood obesity is a significant global health issue (1). Overweight and obesity among young people in China have increased significantly in the last 20 years with changes in dietary patterns and lack of physical exercise (2, 3). Metabolic syndrome (MetS)—a cluster of central obesity, dyslipidemia, hyperglycemia, and hypertension—is increasingly reported in children (4). Adolescents with MetS are at increased risk of developing type 2 diabetes and cardiovascular disease in adulthood, highlighting the importance of early diagnosis (5).

Body mass index (BMI) is the most popular obesity screening method, but it has limited ability to differentiate between fat and lean mass and to selectively identify visceral adipose tissue (6). Instead of total adiposity, visceral adiposity is the cause of metabolic complications due to pro-inflammatory adipokine release and insulin resistance (IR) (7). Waist circumference (WC) enhances the measurement of abdominal adiposity but does not adjust for height, which may misclassify growing children (8). Indices that include WC and height might be more indicative of visceral fat distribution and cardiometabolic risk (9).

Thomas et al. (10) suggested the Body Roundness Index (BRI), a height-adjusted geometric index that estimates body fat and visceral adipose content based on body roundness. BRI has been compared to dual-energy X-ray absorptiometry (DXA) and magnetic resonance imaging (MRI) in adults (10). There is growing evidence that BRI is a better predictor of cardiovascular disease, diabetes, and mortality in adult cohorts than traditional indices, including BMI and WC (11, 12). BRI also showed better detection of IR, hypertension, and dyslipidemia in different populations (13, 14).

Evaluating the dose–response relationship is important because a linear odds ratio alone may not capture BRI’s full risk profile. While adult evidence indicates that cardiometabolic risk accelerates beyond a distinct BRI threshold, it remains unclear whether adolescents exhibit a similar threshold effect (11–14). In the EMSNGS study, Hu et al. (15) found that BRI outperformed traditional obesity measures for cardiometabolic risk assessment in Chinese children. Zhou et al. (16) reported a BRI–hypertension relationship in a pediatric NHANES cohort, and Zhu et al. (17) reported the longitudinal predictive validity of BRI for hypertension in Chinese children. Nevertheless, no research has evaluated the association between BRI and MetS in Chinese adolescents using both linear and non-linear dose–response models, and the biological mechanism linking BRI to MetS has not been systematically interrogated using serum biomarkers.

Metabolic syndrome (MetS) is a complex trait driven by central obesity, involving a network of adipokines, inflammatory mediators, and metabolic regulators (18–22). To capture this trait, we measured 18 serum biomarkers spanning three functional categories, classified *a priori* by their dominant biological roles: (i) energy metabolism and appetite regulation [leptin, irisin, obestatin, neuropeptide Y (NPY), agouti-related protein (AgRP), and visfatin] (20–25); (ii) glucose/lipid metabolism and insulin resistance [resistin, adipocyte fatty acid-binding protein (AFABP), retinol-binding protein 4 (RBP4), lipocalin-2, C1q/TNF-related protein 9 (CTRP9), and secreted frizzled-related protein 5 (SFRP5)] (22, 26–31); and (iii) inflammation and cardiovascular risk [tumor necrosis factor-α (TNF-α), high-sensitivity C-reactive protein (hs-CRP), pentraxin-3, plasminogen activator inhibitor-1 (PAI-1), apolipoprotein B48 (ApoB48), and homocysteine] (20, 32–34). These markers span the principal axes linking visceral adiposity to MetS and enable network and mediation analyses of the BRI–MetS association.

Accordingly, this study aimed to (i) quantify the association between BRI and MetS in Chinese adolescents using both domestic and international criteria; (ii) characterize the dose–response shape of this association and identify a risk-acceleration threshold; (iii) compare the predictive performance of BRI with that of BMI, overall and across sex- and BMI-stratified subgroups; and (iv) explore the serum biomarker networks and two-stage mediation pathways linking BRI to MetS components.

## 2 Methods

### 2.1 Study design and participants

This analysis builds on our previous cross-sectional survey of urban secondary schools in Liaoyang, China (35). Eligible students completed questionnaires and physical measurements with parental consent; those with incomplete data (*n* = 22), unavailable/hemolysed fasting blood, self-reported diabetes or chronic diseases requiring long-term medication, or acute febrile illness within 2 weeks were excluded. In total, 936 students completed both questionnaires and physical measurements. After excluding 22 students with incomplete data, 914 adolescents were included in the analysis. Ethical approval was obtained from the institutional committee, and written informed consent was obtained from guardians.

### 2.2 Clinical and anthropometric assessment

Height, weight, WC, and blood pressure were measured using standardized protocols as previously described (35).

### 2.3 Blood collection and biochemical measurements

All participants fasted for ≥10 h overnight, and fasting venous blood was collected between 07:00 and 09:00 the next morning. Blood samples were sent immediately to the laboratory of Liaoyang Diabetes Hospital for centrifugation within 1 h of collection. Fasting plasma glucose (FPG), triglycerides (TG), total cholesterol (TC), high-density lipoprotein cholesterol (HDL-C), aspartate aminotransferase (AST), alanine aminotransferase (ALT), and serum uric acid (SUA) were measured using an Olympus AU system (Olympus 400; Olympus Optical Company, Tokyo, Japan) at the laboratory of Liaoyang Diabetes Hospital. Low-density lipoprotein cholesterol (LDL-C) was further estimated using the Friedewald formula. Hemoglobin A1c (HbA1c) levels were measured by high-performance liquid chromatography using a D-10 Hemoglobin Testing System (Bio-Rad Laboratories, Inc., Hercules, CA) in the central laboratory of Shengjing Hospital of China Medical University. HbA1c values were standardized to the Diabetes Control and Complications Trial (DCCT) assay. Fasting insulin (FINS) was determined by radioimmunoassay (China Institute of Atomic Energy, Beijing, China). Non-HDL-C was calculated as TC-HDL-C.

### 2.4 Serum biomarker measurements

Eighteen additional serum biomarkers were measured in 683 participants at the central laboratory of Shengjing Hospital of China Medical University. Seventeen biomarkers were quantified using commercial ELISA kits: samples and standards were added to antibody-coated microplates, incubated, washed, and developed with TMB after the detection of antibody and enzyme conjugate application; absorbance was read at 450 nm (reference 570 nm), and concentrations were calculated from four-parameter logistic standard curves. Specifically, leptin, resistin, AFABP, RBP4, AgRP, pentraxin-3, lipocalin-2, PAI-1, TNF-α, hs-CRP, and visfatin were measured using kits from R&D Systems (Minneapolis, USA); irisin, obestatin, and NPY from Phoenix Pharmaceuticals (Burlingame, USA); CTRP9 and SFRP5 from Cloud-Clone (Wuhan, China); and ApoB48 from FUJIFILM Wako Shibayagi (Osaka, Japan). Homocysteine was measured separately using a commercial assay kit (Roche Diagnostics, Mannheim, Germany).

### 2.5 Definitions

BMI was weight (kg)/height squared (kg/m^2^). BRI was computed as: BRI = 364.2–365.5 × sqrt[1 − (WC/(2pi))2/(0.5 × height)2], with WC and height in meters (10).

We used two criteria to define MetS. The Chinese pediatric criteria (CN2025) included abdominal obesity (WC ≥ age- and sex-specific 90th percentile) and at least two of: (1) high FPG ≥ 5.6 mmol/L; (2) hypertension (SBP or DBP ≥ age- and sex-specific 95th percentile); (3) high TG ≥ 1.7 mmol/L; (4) low HDL-C < 1.04 mmol/L or high non-HDL-C ≥ 3.3 mmol/L. The International Diabetes Federation (IDF) criteria included abdominal obesity (WC 90th percentile age- and sex-specific) and at least two of: (1) high FPG 5.6 mmol/L or higher; (2) hypertension (SBP 130 or higher or DBP 85 or higher); (3) high TG 1.7 mmol/L or higher; and (4) low HDL-C 1.03 mmol/L or lower (36). We also analyzed the individual metabolic components as separate binary outcomes.

Dyslipidemia was defined as at least one of: (1) elevated TG ≥ 1.7 mmol/L; (2) low HDL-C < 1.04 mmol/L or elevated non-HDL-C ≥ 3.3 mmol/L (CN2025); and (3) low HDL-C < 1.03 mmol/L (IDF).

### 2.6 Statistical analysis

Analyses used Python 3.11 with standard statistical packages. Significance was set at two-sided *p* < 0.05. Participants were grouped by BRI quartiles. Continuous variables were compared using Kruskal–Wallis or ANOVA, categorical variables by chi-squared tests, and trends by Cochran–Armitage. Logistic regression estimated ORs per BRI unit across three models adjusting for demographic and metabolic factors. Non-linearity was assessed via four-knot restricted cubic splines. ROC and DeLong tests compared BRI and BMI. Subgroup analyses were stratified by sex and BMI *Z*-score categories. Hierarchical clustering identified three biomarker co-expression modules. A two-stage mediation analysis (Sobel test, LASSO selection, parallel mediation) examined BRI-MetS pathways, with FDR correction and joint effects testing via likelihood ratio tests, adjusted for age, sex, and family T2DM history. Primary hypothesis-driven analyses (BRI–MetS associations, RCS dose–response, and AUC comparisons) are reported with nominal two-sided *p* < 0.05, whereas FDR correction was applied to the exploratory biomarker comparisons and mediation analyses as described above.

## 3 Results

### 3.1 Baseline characteristics of participants by BRI quartiles

Of the 914 participants, 193 (21.1%) met CN2025 MetS criteria, and 120 (13.1%) met IDF criteria. Participants were stratified into quartiles according to their BRI values (Q1: median 1.80; Q2: 2.37; Q3: 3.05; Q4: 4.13) (Table 1).

**Table 1:** Baseline characteristics of participants by BRI quartiles.

Variable	Q1 (*n* = 229)	Q2 (*n* = 228)	Q3 (*n* = 229)	Q4 (*n* = 228)	*p* value	*p* for trend
BRI	1.80 (1.63, 1.94)	2.37 (2.25, 2.50)	3.05 (2.85, 3.24)	4.13 (3.78, 4.70)	<0.001	<0.001
Age (years)	13.00 (12.00, 15.00)	13.50 (12.00, 15.00)	14.00 (12.00, 15.00)	13.00 (12.00, 15.00)	0.59	0.28
Male, *n* (%)	116 (50.7)	119 (52.2)	113 (49.3)	138 (60.5)	0.07	–
Acanthosis nigricans, *n* (%)	2 (0.9)	7 (3.1)	9 (3.9)	64 (28.1)	<0.001	–
Family history of T2DM, *n* (%)	109 (47.6)	122 (53.5)	130 (56.8)	144 (63.2)	0.009	–
WC (cm)	66.50 (64.00, 69.50)	72.23 ± 4.18	78.66 ± 4.78	89.50 (84.50, 94.50)	<0.001	<0.001
BMI (kg/m^2^)	18.41 ± 1.74	19.73 (18.39, 21.17)	21.68 (19.44, 23.63)	26.34 ± 4.07	<0.001	<0.001
SBP (mmHg)	115.00 (107.00, 124.00)	115.00 (107.00, 122.00)	117.34 ± 12.77	122.50 (112.75, 131.00)	<0.001	<0.001
DBP (mmHg)	72.87 ± 10.77	72.25 ± 10.83	73.20 ± 10.81	73.93 ± 10.98	0.41	0.2
FPG (mmol/L)	4.70 (4.40, 5.00)	4.80 (4.50, 5.10)	4.80 (4.50, 5.00)	4.70 (4.40, 5.10)	0.73	0.47
HDL-C (mmol/L)	1.19 (1.00, 1.35)	1.07 ± 0.25	1.06 (0.88, 1.25)	0.97 (0.80, 1.18)	<0.001	<0.001
Non-HDL-C (mmol/L)	3.15 (2.58, 3.74)	3.25 (2.72, 3.81)	3.46 ± 0.76	3.70 ± 0.82	<0.001	<0.001
TG (mmol/L)	0.89 (0.66, 1.20)	0.84 (0.63, 1.23)	0.94 (0.69, 1.31)	1.17 (0.80, 1.63)	<0.001	<0.001
TC (mmol/L)	4.32 (3.76, 4.86)	4.40 ± 0.79	4.51 ± 0.75	4.68 ± 0.83	<0.001	<0.001
LDL-C (mmol/L)	3.30 (3.25, 3.45)	3.29 (3.24, 3.45)	3.34 (3.26, 3.49)	3.35 (3.26, 3.52)	0.004	<0.001
HOMA-IR	3.17 (2.30, 4.50)	3.63 (2.40, 4.80)	3.88 (2.99, 5.21)	4.50 (3.44, 6.47)	<0.001	<0.001
HbA1c (%)	5.40 ± 0.26	5.40 (5.30, 5.60)	5.50 (5.30, 5.60)	5.50 (5.30, 5.60)	0.08	0.02
MetS and components						
Abdominal obesity, *n* (%)	0 (0.0)	20 (8.8)	164 (71.6)	225 (98.7)	<0.001	–
Elevated FPG, *n* (%)	9 (3.9)	17 (7.5)	16 (7.0)	18 (7.9)	0.31	–
Elevated TG, *n* (%)	33 (14.4)	24 (10.5)	34 (14.8)	71 (31.1)	<0.001	–
Low HDL-C or elevated nonHDL-C (CN2025), *n* (%)	106 (46.3)	133 (58.3)	143 (62.4)	171 (75.0)	<0.001	–
Low HDL-C (IDF), *n* (%)	70 (30.6)	105 (46.1)	107 (46.7)	132 (57.9)	<0.001	–
Hypertension (CN2025), *n* (%)	84 (36.7)	73 (32.0)	94 (41.0)	116 (50.9)	<0.001	–
Hypertension (IDF), *n* (%)	47 (20.5)	50 (21.9)	59 (25.8)	87 (38.2)	<0.001	–
MetS (CN2025), *n* (%)	0 (0.0)	7 (3.1)	63 (27.5)	123 (53.9)	<0.001	–
No. of MetS components (CN2025)	1.00 (0.00, 1.00)	1.00 (1.00, 2.00)	1.97 ± 0.98	2.64 ± 0.90	<0.001	<0.001
MetS (IDF), *n* (%)	0 (0.0)	4 (1.8)	33 (14.4)	83 (36.4)	<0.001	–
No. of MetS components (IDF)	1.00 (0.00, 1.00)	1.00 (0.00, 1.00)	1.59 ± 0.90	2.00 (2.00, 3.00)	<0.001	<0.001

Data are presented as median (IQR) for non-normally distributed variables or mean ± SD for normally distributed variables. Categorical variables are shown as *n* (%). BRI, Body Roundness Index; WC, waist circumference; BMI, body mass index; SBP, systolic blood pressure; DBP, diastolic blood pressure; FPG, fasting plasma glucose; HDL-C, high-density lipoprotein cholesterol; TG, triglycerides; TC, total cholesterol; LDL-C, low-density lipoprotein cholesterol; HOMA-IR, homeostatic model assessment of insulin resistance; HbA1c, glycated hemoglobin; MetS, metabolic syndrome. CN2025 denotes the Chinese pediatric MetS criteria (2025); IDF denotes the International Diabetes Federation pediatric criteria. *p* values were derived from Kruskal–Wallis test or one-way ANOVA for continuous variables and chi-square test for categorical variables. *p* for trend was assessed using linear regression for continuous variables. “–” indicates not applicable for categorical variables.

There were no differences in age and sex distributions between quartiles (*p* = 0.59 and *p* = 0.07). WC, BMI, and SBP all monotonically increased with BRI (all *p*-trend <0.001); DBP did not vary (*p* = 0.41). Higher BRI quartiles had a higher frequency of acanthosis nigricans and a family history of T2DM.

Metabolic profiles worsened with increasing BRI quartiles. HDL-C fell from 1.19 mmol/L (Q1) to 0.97 mmol/L (Q4) (*p*-trend <0.001); non-HDL-C, TG, TC, and LDL-C all increased (all *p*-trend <0.01). FPG and HbA1c were stable across groups (*p* = 0.73, *p* = 0.08). HOMA-IR rose from 3.17 (Q1) to 4.50 (Q4) (*p*-trend <0.001), indicating progressive IR.

MetS prevalence increased sharply across quartiles: CN2025, 0–53.9% (*p* < 0.001); IDF, 0–36.4% (*p* < 0.001). There was also a significant increase in the proportion with dyslipidemia and hypertension (all *p* < 0.001); the proportion with elevated FPG did not increase (*p* = 0.31).

### 3.2 Association of BRI with MetS and its metabolic components

Each one-unit BRI increase was associated with elevated MetS odds: CN2025 OR = 3.63 (95%CI 2.91–4.53, *p* < 0.001) and IDF OR = 2.64 (95%CI 2.14–3.26, *p* < 0.001) in fully adjusted models. BRI was also associated with dyslipidemia (elevated TG: OR = 1.43, 95% CI: 1.20–1.71; low HDL-C/elevated non-HDL-C under CN2025: OR = 1.65, 95% CI: 1.41–1.94; low HDL-C under IDF: OR = 1.48, 95% CI: 1.28–1.72) and hypertension (CN2025: OR = 1.27, 95% CI: 1.11–1.46; IDF: OR = 1.31, 95% CI: 1.13–1.51). There was no linear correlation with high FPG (Supplemenatry Table S1).

Non-linear BRI–MetS relationships were verified by RCS analysis (Figure 1A) for both criteria (overall *p* < 0.001), where the risk escalated sharply above BRI of approximately 4.0 (non-linearity *p* < 0.001). The BRI ≈ 4.0 threshold from dose–response curves showed high specificity (~91–93%) but low-to-moderate sensitivity (0.39–0.45) for MetS, reflecting its role in identifying sharply accelerating risk rather than serving as an optimal classification threshold. By contrast, the Youden J-optimal cutoffs maximizing accuracy were 2.91 (bootstrap 95% CI 2.63–3.08) for CN2025 and 2.77 (2.60–3.39) for IDF, which may be more appropriate for screening.

**Figure 1:**
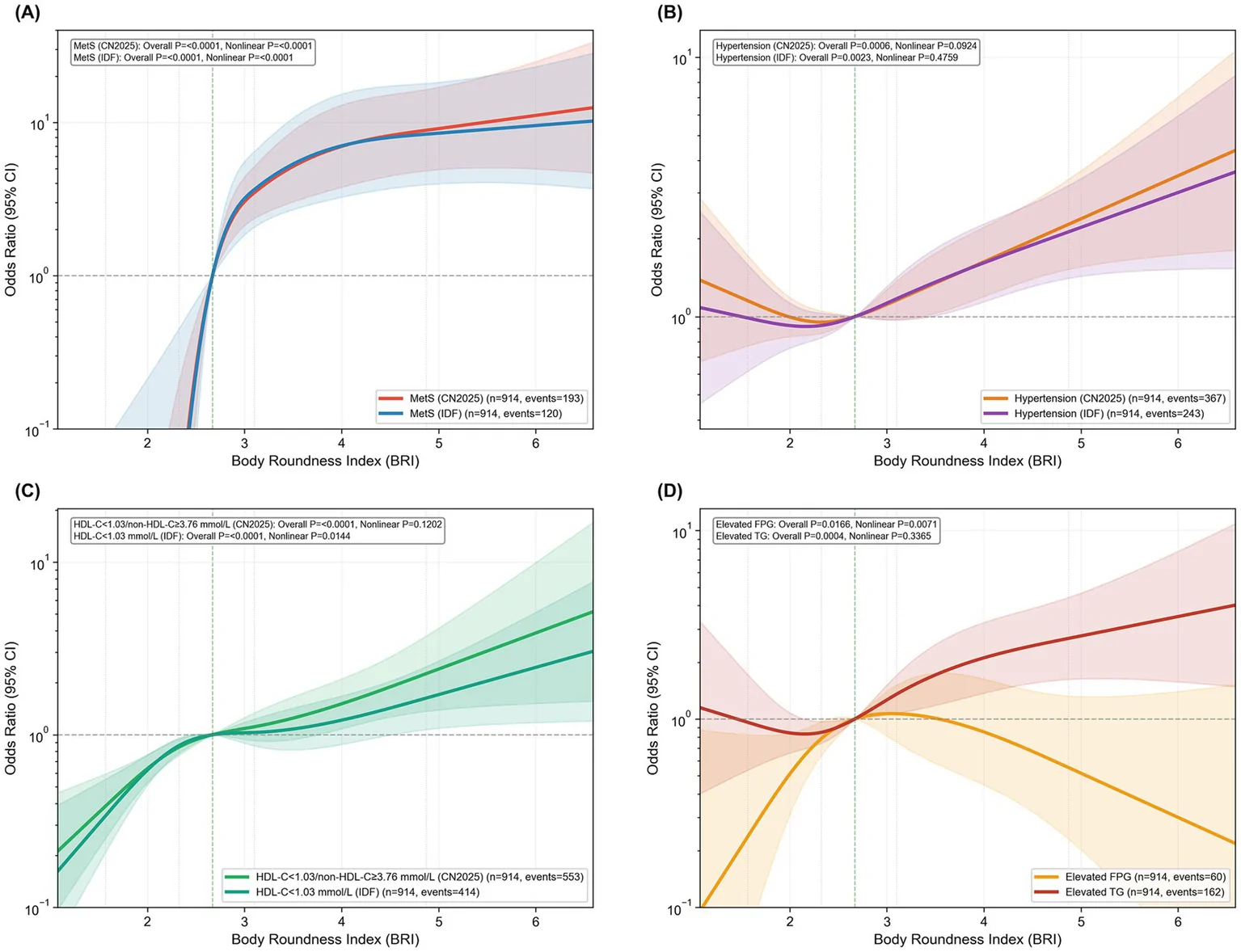
Dose–response associations of BRI with MetS and metabolic components (*n* = 914). Four-knot RCS logistic regression models adjusted for age, sex, ALT, AST, and T2DM family history (Model 3). Odds ratios (log scale) with 95% CIs (shaded) are shown; reference lines: Horizontal dashed = OR 1, dotted = knots, green dashed = median BRI (2.67). *p*-values for overall and non-linear trends (Wald tests) appear in each panel. The BRI ≈ 4.0 point of steepest OR increase indicates the risk-acceleration threshold. **(A)** MetS by CN2025 (193 events) and IDF (120 events). **(B)** Hypertension by CN2025 (367 events) and IDF (243 events). **(C)** Dyslipidemia by CN2025 (HDL-C < 1.03 or non-HDL-C ≥ 3.76 mmol/L; 553 events) and IDF (HDL-C < 1.03 mmol/L; 414 events). **(D)** Elevated FPG (60 events) and elevated TG (162 events).

There were significant dose–response relationships between hypertension and dyslipidemia (overall *p* < 0.001) (Figures 1B,C). Interestingly, the significant overall relationship between BRI and FPG was observed with higher FPG, which did not show any linear relationship in logistic regression, but in the spline analysis (overall *p* = 0.017, non-linearity *p* = 0.007), indicating a threshold effect that was not observed with conventional logistic regression (Figure 1D).

Taken together, the RCS analyses showed that the odds of MetS increased non-linearly with BRI, rising modestly at low BRI values and accelerating sharply beyond approximately BRI ≈ 4.0, with a similar pattern for hypertension and dyslipidemia.

### 3.3 Comparative predictive performance of BRI vs. BMI for MetS

The fully adjusted Model 3 (Figure 2A; Supplemenatry Table S2) ROC comparisons revealed that BRI was significantly better than BMI in defining MetS using the CN2025 criteria (AUC 0.873 vs. 0.835; deltaAUC = 0.038, *p* = 0.005). BRI also exhibited a numerically higher AUC (0.859 vs. 0.846), but this was not statistically significant (*p* = 0.309) for MetS defined by the IDF criteria.

**Figure 2:**
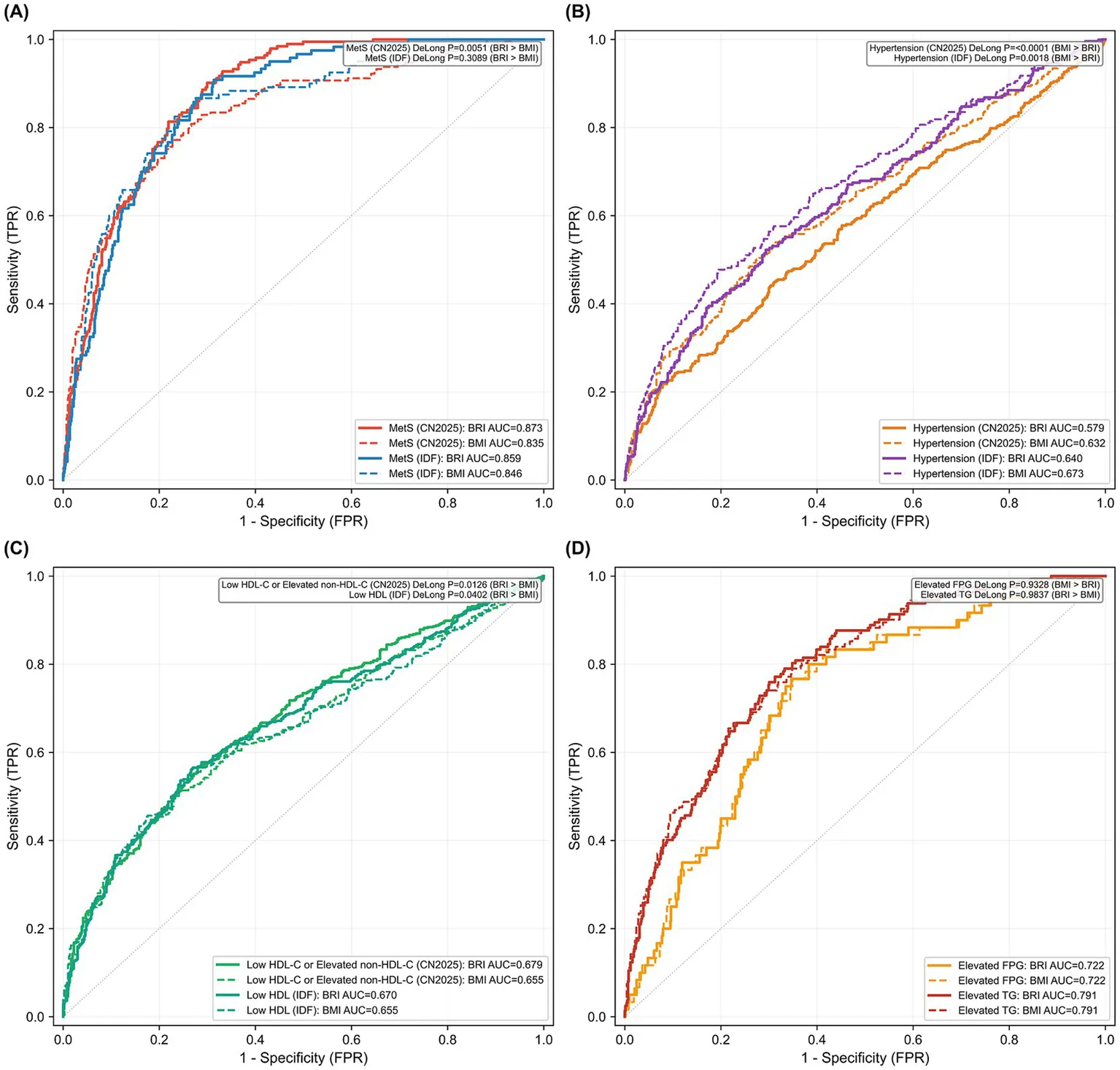
ROCs compare BRI and BMI for predicting MetS and its metabolic components. Model 3 AUCs (age, sex, ALT, AST, and T2DM family history) with 95% CIs; DeLong *p*-values (BRI vs. BMI). Dashed diagonal = no discrimination. **(A)** MetS (CN2025/IDF). **(B)** Hypertension (CN2025/IDF). **(C)** Low HDL-C or elevated non-HDL-C (CN2025). **(D)** Elevated FPG and elevated TG.AUCs near 1 = better discrimination; significant *p*-values indicate BRI vs. BMI differences.

For the metabolic components, BRI significantly outperformed BMI in predicting Low HDL-C or elevated non-HDL-C (CN2025) (AUC: 0.679 vs. 0.655, ΔAUC = +0.023, *p* = 0.013) and Low HDL (IDF) (AUC: 0.670 vs. 0.655, ΔAUC = +0.015, *p* = 0.040) (Figure 2B). However, BMI was significantly superior to BRI for predicting hypertension (Figure 2C), with a wider AUC gap under CN2025 (AUC: 0.632 vs. 0.579, ΔAUC = −0.053, *p* < 0.001) than under IDF criteria (AUC: 0.673 vs. 0.640, ΔAUC = −0.032, *p* = 0.002). In the case of high FPG and high TG, the two indices had almost the same predictive performance (Figure 2D). BRI had the best predictive accuracy of MetS based on CN2025 criteria (AUC = 0.873), with a sensitivity of 90.2% and specificity of 70.0%.

### 3.4 Subgroup analysis of BRI–MetS association

To determine whether the effect of BRI on MetS varied by subgroups, we stratified participants by sex and by BMI category (Table 2).

**Table 2:** Subgroup analysis of BRI predicting MetS and its metabolic components.

Subgroup	Outcome	*N*	Events	OR	95% CI	*p* value
Sex						
Male	MetS (CN2025)	486	118	3.8224	2.8143–5.1914	<0.001
	MetS (IDF)	486	82	2.6964	2.0578–3.5332	<0.001
	Elevated FPG	486	37	0.9472	0.6908–1.2987	0.736
	Elevated TG	486	86	1.7484	1.3813–2.2129	<0.001
	Hypertension (CN2025)	486	205	1.3178	1.1030–1.5745	0.002
	Hypertension (IDF)	486	155	1.3364	1.1107–1.6079	0.002
	HDL < 1.03 or non-HDL > =3.76 (CN2025)	486	287	1.9345	1.5471–2.4189	<0.001
	HDL < 1.03 (IDF)	486	221	1.7589	1.4357–2.1549	<0.001
Female	MetS (CN2025)	428	75	3.4871	2.5116–4.8417	<0.001
	MetS (IDF)	428	38	2.6772	1.8633–3.8468	<0.001
	Elevated FPG	428	23	1.2976	0.8447–1.9933	0.234
	Elevated TG	428	76	1.0895	0.8160–1.4547	0.561
	Hypertension (CN2025)	428	162	1.1947	0.9665–1.4769	0.1
	Hypertension (IDF)	428	88	1.2783	1.0020–1.6307	0.048
	HDL < 1.03 or non-HDL > =3.76 (CN2025)	428	266	1.34	1.0605–1.6931	0.014
	HDL < 1.03 (IDF)	428	193	1.2124	0.9779–1.5031	0.079
BMI						
Normal weight	MetS (CN2025)	764	97	3.4842	2.5937–4.6804	<0.001
	MetS (IDF)	764	50	2.6211	1.8860–3.6428	<0.001
	Elevated FPG	764	49	1.3798	0.9653–1.9724	0.077
	Elevated TG	764	108	1.2199	0.9341–1.5931	0.144
	Hypertension (CN2025)	764	267	0.9157	0.7573–1.1073	0.364
	Hypertension (IDF)	764	166	0.9445	0.7587–1.1757	0.609
	HDL < 1.03 or non-HDL > =3.76 (CN2025)	764	442	1.6316	1.3304–2.0009	<0.001
	HDL < 1.03 (IDF)	764	326	1.4786	1.2174–1.7960	<0.001
Overweight	MetS (CN2025)	98	56	1.7088	0.8544–3.4175	0.13
	MetS (IDF)	98	41	1.9451	1.0064–3.7596	0.048
	Elevated FPG	98	9	0.3994	0.0986–1.6179	0.198
	Elevated TG	98	32	0.8645	0.4251–1.7580	0.688
	Hypertension (CN2025)	98	59	1.0237	0.5699–1.8388	0.938
	Hypertension (IDF)	98	42	1.3317	0.7189–2.4668	0.362
	HDL < 1.03 or non-HDL > =3.76 (CN2025)	98	68	2.3328	1.0719–5.0767	0.033
	HDL < 1.03 (IDF)	98	52	1.7956	0.9441–3.4151	0.074
Obese	MetS (CN2025)	52	40	1.3711	0.5656–3.3236	0.485
	MetS (IDF)	52	29	0.7495	0.3933–1.4282	0.381
	Elevated FPG	52	2	–	–	–
	Elevated TG	52	22	0.7954	0.3217–1.9670	0.62
	Hypertension (CN2025)	52	41	1.269	0.5409–2.9775	0.584
	Hypertension (IDF)	52	35	0.7657	0.3793–1.5458	0.456
	HDL < 1.03 or non-HDL > =3.76 (CN2025)	52	43	1.162	0.4663–2.8957	0.747
	HDL < 1.03 (IDF)	52	36	0.8888	0.4201–1.8803	0.758

Logistic regression models were used to estimate odds ratios (OR) per one-unit increase in BRI. Sex-stratified models were adjusted for age, ALT, AST, and family history of T2DM. BMI-stratified models were adjusted for age, sex, ALT, AST, and family history of T2DM. BMI categories were defined using ZBMI scores based on Chinese adolescent reference standards: normal weight (ZBMI < 1), overweight (ZBMI ≥1 to <2), and obese (ZBMI ≥2). OR: odds ratio. CI: confidence interval. “–” indicates insufficient events (<5) or model non-convergence.

In male adolescents, BRI was significantly associated with MetS under both CN2025 (OR = 3.82, 95% CI: 2.81–5.19, *p* < 0.001) and IDF criteria (OR = 2.70, 95% CI: 2.06–3.53, *p* < 0.001). BRI was also significantly associated with dyslipidemia and hypertension. In female adolescents, BRI was also significantly associated with MetS (CN2025: OR = 3.49, 95% CI: 2.51–4.84; IDF: OR = 2.68, 95% CI: 1.86–3.85; both *p* < 0.001) and with low HDL-C/elevated non-HDL-C under CN2025 criteria (OR = 1.34, 95% CI: 1.06–1.69, *p* = 0.014). Nevertheless, the relationships with high TG (OR = 1.09, *p* = 0.561), hypertension according to CN2025 criteria (OR = 1.19, *p* = 0.100), and low HDL-C according to IDF criteria (OR = 1.21, *p* = 0.079) were not statistically significant in female adolescents, indicating that the metabolic impacts of BRI on lipid and blood pressure components might be less significant in female adolescents than in male adolescents.

The BRI-MetS association was greatest in normal-weight participants (*n* = 764). In this subgroup, each one-unit increase in BRI was associated with 3.48-fold higher odds of MetS under CN2025 criteria (95% CI: 2.59–4.68, *p* < 0.001) and 2.62-fold higher odds under IDF criteria (95% CI: 1.89–3.64, *p* < 0.001). Dyslipidemia was also found to be significantly associated with BRI in normal-weight adolescents with the exception of high TG. BRI was not associated with hypertension in normal-weight adolescents, which suggests that BRI reflects metabolic risk regardless of overall body weight.

In the overweight subgroup (*n* = 98), BRI remained significantly associated with MetS under IDF criteria (OR = 1.95, 95% CI: 1.01–3.76, *p* = 0.048) and with low HDL-C or elevated non-HDL-C under CN2025 (OR = 2.33, 95% CI: 1.07–5.08, *p* = 0.033), whereas the association with MetS under CN2025 criteria was not significant. No meaningful correlations were found in obese participants (*n* = 52), which was probably due to the small sample size and low BRI variability.

BRI was the strongest predictor of MetS in normal-weight and overweight adolescents, with significant sex differences in lipid and blood pressure correlations.

### 3.5 Biomarker networks and co-expression modules across BRI quartiles

UA, FINS, and ALT varied significantly among BRI quartiles (all *p* < 0.001) with increasing concentrations; SFRP5 exhibited a non-monotonic pattern (*p* = 0.006) (Figure 3; Supplementary Figure S1; Supplementary Table S3). Hierarchical clustering revealed three co-expression modules (Figure 4A):

**Figure 3:**
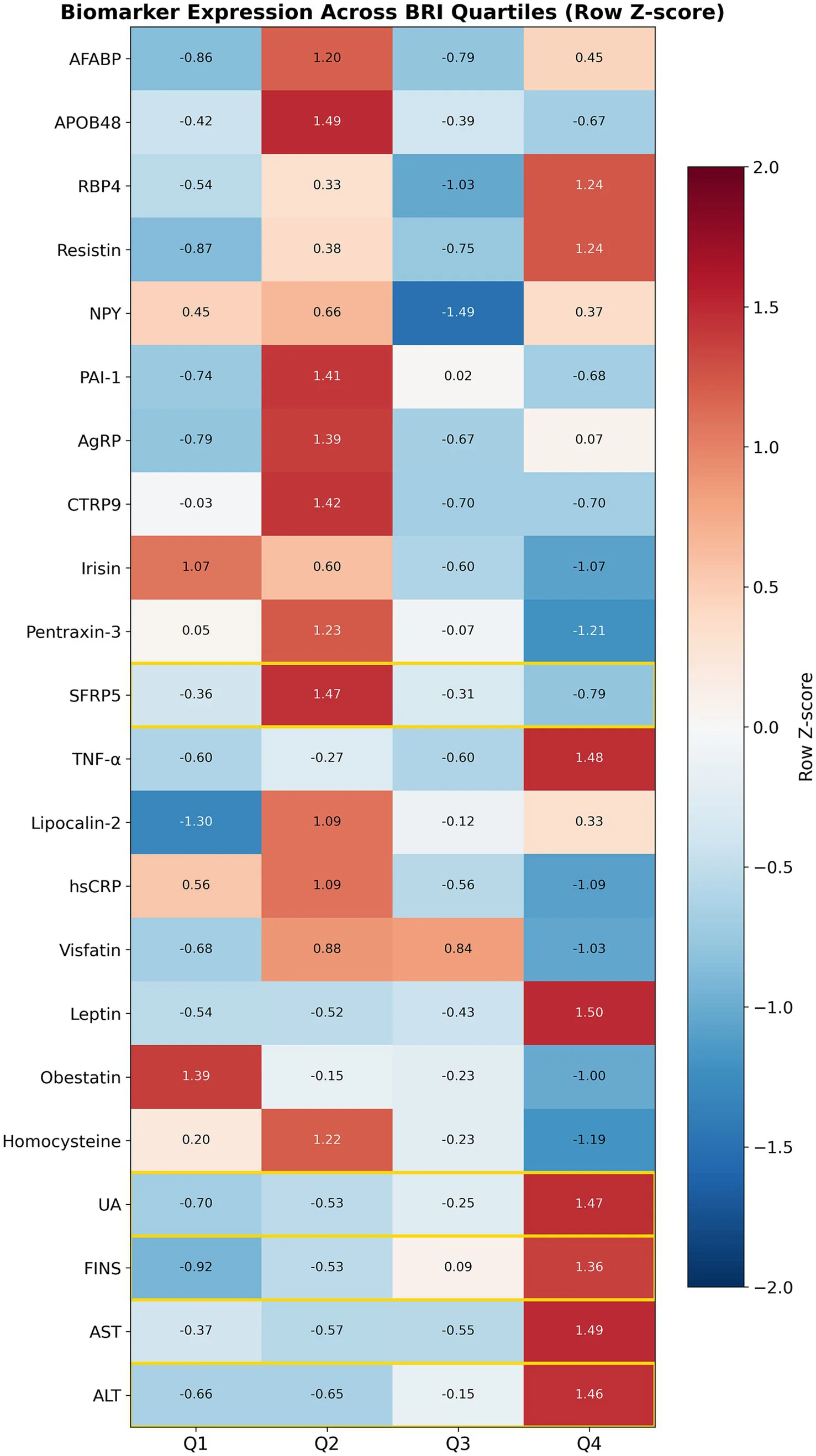
Heatmap of biomarker expression across BRI quartiles. Rows: 22 serum biomarkers; Columns: BRI quartiles (Q1–Q4). Cell colors denote row-standardized *Z*-scores (blue = below, red = above mean; scale −2 to +2), with values to two decimals. Gold-framed biomarkers (FINS, ALT, UA, and SFRP5) differed significantly across quartiles after FDR correction (Kruskal–Wallis).

**Figure 4:**
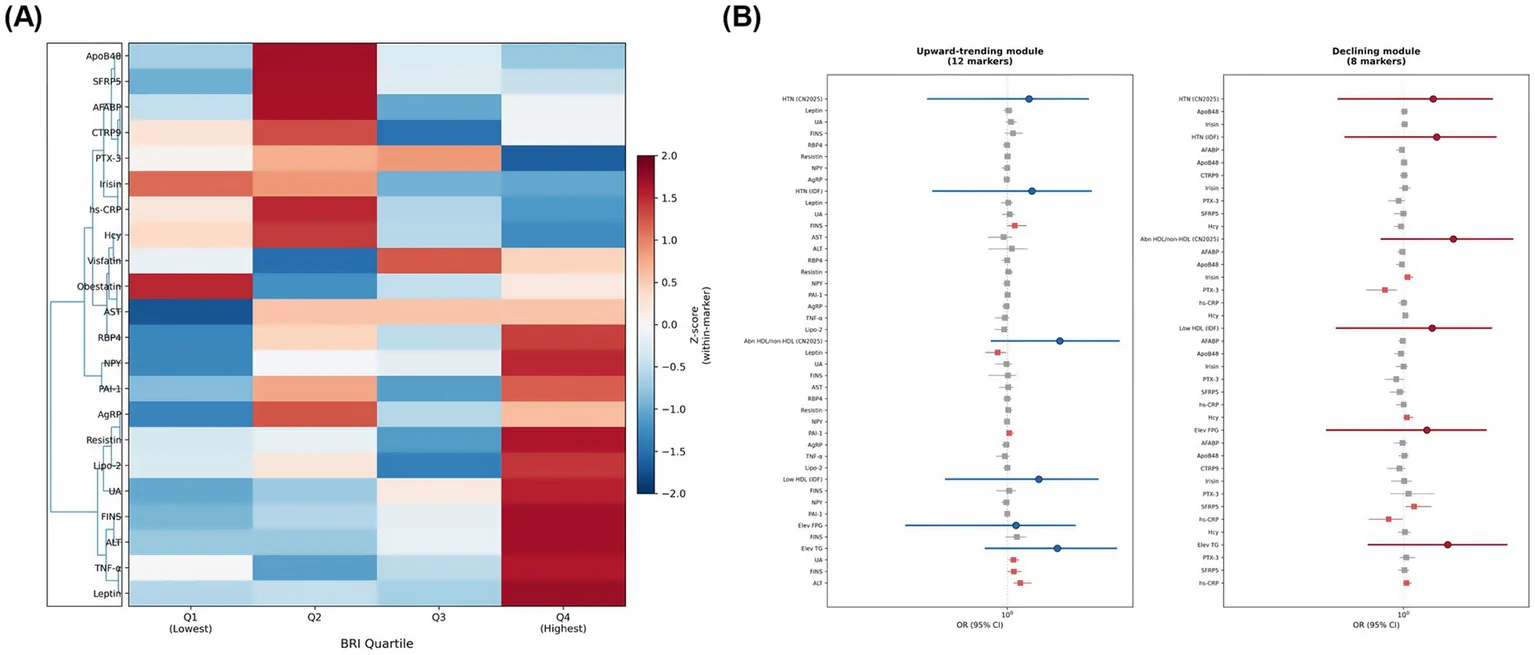
Serum biomarkers as mediators of the BRI–MetS association. **(A)** Clustering of 22 biomarkers across BRI quartiles identified three modules (up/down/flat). **(B)** Parallel mediation (BRI → MetS). LASSO-selected mediators; effects estimated (logistic/linear, adjusted for age/sex/T2DM) with bootstrap CIs. Likelihood ratio tests for joint effects; FDR-corrected within modules. Only FDR-*p* < 0.05 sets shown. PM (%) = proportion mediated. Analyses are exploratory/hypothesis-generating.

Upward module (12 markers): Leptin, UA, FINS, AST, ALT, RBP4, Resistin, NPY, PAI-1, AgRP, TNF-alpha, and Lipocalin-2; these increased from Q1 to Q4, with the peak change from Q3 to Q4.

Declining module (eight markers): AFABP, ApoB48, CTRP9, Irisin, Pentraxin-3, SFRP5, hs-CRP, and homocysteine. These reached their highest levels in Q1/Q2 and decreased with increasing levels of BRI, indicating a gradual depletion of a protective biomarker signature.

Flat module (2 markers): Visfatin and Obestatin did not exhibit any evident quartile trends.

### 3.6 Joint mediation effects of biologically related biomarker clusters

Upward and declining modules of LASSO-selected mediators were included in parallel mediation models with BRI as exposure and each MetS component as outcome (Figure 4B; Supplementary Table S4). Comparisons between participants with and without complete biomarker data are summarized in Supplementary Table S5. Participants lacking biomarker data were slightly older [median 14 (IQR 13–15) vs. 13 (12–15) years, *p* < 0.001], had a slightly higher waist circumference [77.5 (69.8–85.5) vs. 74.5 (69.5–81.5) cm, *p* = 0.016], and a higher prevalence of IDF-defined MetS (17.3% vs. 11.7%, *p* = 0.039).

The most significant and consistent mediation effects were observed in the upward module. In the case of elevated TG, three LASSO-selected mediators (UA, FINS, and ALT) mediated 52.9% of the BRI effect (combined IE = 0.265; total OR 1.650 and direct OR 1.281). All three showed significant indirect effects: ALT (IE = 0.132, PM = 26.3%, *p* < 0.001), FINS (IE = 0.070, PM = 13.9%, *p* = 0.021), and UA (IE = 0.064, PM = 12.7%, *p* = 0.005). For hypertension, the upward module mediated 54.1% of BRI effects under CN2025 criteria (combined IE = 0.118, PM = 54.1%, LR *p* = 0.008) and 59.5% under IDF criteria (combined IE = 0.148, PM = 59.5%, LR *p* = 0.037). FINS alone mediated the IDF hypertension path (IE = 0.077, PM = 31.1%, *p* = 0.037). In the case of high FPG, FINS alone was nearly completely mediated (IE = 0.097, PM = 109.2, *p* = 0.055), indicating that the BRI–FPG relationship is mediated by IR. For HDL/non-HDL abnormality (CN2025), net upward mediation was minimal (combined PM = 0.9%), with opposing effects: Leptin (IE = −0.037, PM = −7.1%, *p* = 0.019) vs. PAI-1 (IE = 0.022, PM = 4.2%, *p* = 0.038). There was no significant low HDL (IDF) mediation (PM = 7.0, all *p* > 0.05).

Declining module mediated fewer outcomes. For elevated FPG, SFRP5 was the strongest individual mediator (IE = 0.108, PM = 46.3%, *p* = 0.005); hsCRP showed a negative indirect effect (IE = −0.059, PM = −25.2%, *p* = 0.032), yielding combined PM = 50.7%. For HDL/non-HDL abnormality (CN2025), Irisin (IE = 0.041, PM = 8.2%, *p* = 0.007) and Pentraxin-3 (IE = −0.075, PM = −15.0%, *p* < 0.001) showed opposing effects (combined PM = −3.6%). Homocysteine mediated low HDL under IDF criteria (IE = 0.036, PM = 12.6%, *p* = 0.047). The hsCRP-mediated high TG in this module (IE = 0.031, PM = 6.9, *p* = 0.033), which validates an inflammation pathway independent of upward module metabolism markers. There was no declining module mediation of hypertension.

## 4 Discussion

In 914 Chinese adolescents aged 11–16 years, each one-unit increase in BRI was associated with 3.63-fold higher odds of MetS by the CN2025 criteria (95% CI 2.91–4.53) and 2.64-fold higher odds by the IDF criteria (95% CI 2.14–3.26). The dose–response analysis showed that MetS risk increased non-linearly with BRI and accelerated sharply beyond a BRI of approximately 4.0. BRI outperformed BMI in discriminating MetS (delta AUC + 0.038, *p* = 0.005), particularly among normal-weight adolescents, and network and mediation analyses identified distinct biomarker modules and pathways (notably the UA–FINS–ALT axis) through which BRI may act. These findings indicate that BRI is a clinically useful, non-linear risk marker of adolescent MetS and point to specific biological mechanisms that warrant prospective evaluation.

BRI has generally outperformed BMI and WC in predicting cardiovascular disease and mortality in adults (11–13), but there is limited evidence in pediatrics. Our results confirm and expand recent pediatric reports. In line with Hu et al. (15), BRI outperformed traditional obesity indices in predicting cardiometabolic risk in Chinese youth. This is in line with Zhou et al. (16) and Zhu et al. (17), who reported BRI cross-sectional and longitudinal associations between BRI and hypertension, respectively. Recent evidence beyond the Chinese context supports our findings: in U.S. children and adolescents aged 8–17 years, BRI outperformed BMI in predicting cardiometabolic risk (37), and a 2025 systematic review and meta-analysis confirmed BRI’s predictive value for NAFLD (pooled OR = 2.87; AUC = 0.803) (38). However, dedicated BRI studies in other Asian adolescent populations remain limited, underscoring the need for future multi-population validation. It is important to note that current research shows that BRI can detect a high-risk phenotype in normal-weight adolescents-individuals who would otherwise not be detected by BMI-based screening. BRI requires only routine measurements (waist and height) and minimal training, enabling easy integration into school screening to complement BMI-based assessment, especially for detecting central adiposity in normal-weight children. Before routine use, population- and context-specific cutoffs, prospective validation, and cost-effectiveness analyses are required.

RCS found a BRI threshold (~4.0) above which MetS risk increased exponentially, consistent with the pathological role of visceral fat (4, 7). FPG did not have a linear BRI relationship, but spline analysis indicated a non-monotonic relationship (*p* = 0.017). This trend is consistent with near-total FINS mediation (109.2%), indicating that central obesity mediates hyperglycemia via IR rather than direct effects, and that frank FPG elevation likely indicates beta-cell decompensation at high BRI levels.

Sex differences were found: BRI-TG and BRI-hypertension associations were significant in male adolescents but not for female adolescents. This is reflective of established patterns of pubertal fat distribution-higher visceral fat in male adolescents and subcutaneous fat in female adolescents (4, 39). BMI was more effective than BRI in predicting hypertension, perhaps because BMI is a more accurate measure of total mechanical vascular load, whereas BRI is a more specific measure of dyslipidemia caused by visceral fat (40). BRI’s best performance in normal-weight adolescents has clinical implications—including that BRI in school screening would help detect metabolically high-risk normal-weight youth not detected by BMI.

The analysis of biomarker networks showed dynamic restructuring of serum profiles at BRI levels. The upward module (leptin, UA, FINS, ALT, AST, RBP4, resistin, NPY, PAI-1, AgRP, TNF-α, and lipocalin-2) comprises markers of adiposity-driven insulin resistance, hepatic stress, and systemic inflammation; their concordant rise with BRI is consistent with the concept of an integrated “metabolic-inflammatory” burden. The declining module (AFABP, ApoB48, CTRP9, irisin, pentraxin-3, SFRP5, hs-CRP, and homocysteine) includes markers with protective or context-dependent roles; irisin promotes white-adipose browning and energy expenditure (20, 21), and SFRP5 is an anti-inflammatory adipokine (22), and their depletion at high BRI may represent loss of protective feedback. These markers are not captured by conventional anthropometry or routine biochemistry, and their module-level behavior supports multi-marker profiling rather than single-marker assessment in pediatric metabolic risk evaluation.

The simultaneous increase in the UA-ALT-FINS triad in Q4 indicates the presence of synergistic metabolic deterioration with hyperuricemia, liver stress, and IR. Mediation analysis showed that the combination of these three markers accounts for more than half of the BRI triglyceride effect, which is in line with recent pediatric data (41–44). Near-complete FINS mediation of BRI-FPG (109.2%) highlights IR as the main mechanistic mediator between central obesity and glucose dysregulation.

Protective biomarkers (irisin, SFRP5, and CTRP9) were lower in participants with central obesity. Irisin, which is involved in white adipose browning and energy expenditure (20), was lower in adolescents with MetS (21). In the current research, BRI-HDL/non-HDL abnormality (8.2%) was partially mediated by irisin. SFRP5 was the most prevalent FPG mediation (PM = 46.3%), which is in line with its known role in pediatric IR (22). The presence of divergent pentraxin-3 (negative) and irisin (positive) on HDL effects exemplifies dualistic network dynamics, favoring multi-marker over single-marker profiling.

This is the first study to thoroughly evaluate BRI as a predictor of MetS among Chinese adolescents, using both linear and non-linear frameworks with detailed mediator analyses. Dual MetS definitions (CN2025, IDF) enhance cross-study comparability. RCS captured non-linear patterns that logistic regression missed. Integrating clustering and module-based parallel mediation provided a systems-level perspective on the BRI-MetS pathway.

There are several limitations worth considering. The cross-sectional design does not allow causal inference; therefore, the mediation analyses are exploratory and hypothesis-generating rather than confirmatory, and the identified pathways require validation in longitudinal studies. Of the 914 participants, 683 (74.7%) had biomarker data. Missingness was associated with older age and higher IDF-MetS prevalence, but not BMI/BRI/CN2025-MetS. Despite adjustment for age and sex, potential selection bias remains, limiting generalizability. Spanning ages 11–16 years (the pubertal transition), our cohort lacked Tanner stage data. Pubertal timing may partly explain sex-specific findings (e.g., stronger BRI–TG/hypertension associations in boys) given puberty’s effects on body composition and fat distribution. Future studies should include Tanner staging.

## 5 Conclusion

BRI is a strong, non-linear predictor of MetS in Chinese adolescents and performed better than BMI, particularly for identifying dyslipidemia and risk among normal-weight adolescents. Sex-specific associations endorse the need for customized screening strategies. A BRI cutoff of 4.0 represents a clinically actionable threshold. Network and mediation analyses reveal the ALT-FINS-UA axis as the main pathway linking BRI to hypertriglyceridemia and hypertension, whereas protective adipokines (irisin and SFRP5) were involved in the glycemic and HDL pathways. Longitudinal studies and intervention trials are required to determine whether these mediators are viable therapeutic targets and whether BRI-guided screening can enhance cardiometabolic outcomes in adolescents.

## Data Availability

The raw data supporting the conclusions of this article will be made available by the authors, without undue reservation.

## Supplementary material

SUPPLEMENTARY FIGURE S1 Biomarkers significantly different across BRI quartiles. Box plots of FINS, ALT, UA, and SFRP5 across BRI quartiles (Q1–Q4). ^*^*p* < 0.05, *p* < 0.01, **p* < 0.001.
