# Supplementary material for: Body roundness index and adolescent metabolic syndrome: dose–response analysis and serum biomarker mediation
Source: Front Nutr. 2026 Sep 16;13:1930822. doi: 10.3389/fnut.2026.1930822 (PMC13623853; doi:10.3389/fnut.2026.1930822)
Supplement: Supplementary file 1 [file Supplementary_file_1.docx]

**Table S1. Multivariable logistic regression of BRI for MetS and its metabolic components.**

| **Outcome** | **Model1** |  | **Model2** |  | **Model3** |  | **Events** |
| --- | --- | --- | --- | --- | --- | --- | --- |
| MetS (CN2025) | 3.87 (3.14–4.78) | <0.001 | 3.89 (3.15–4.81) | <0.001 | 3.63 (2.91–4.53) | <0.001 | 193 |
| MetS (IDF) | 2.93 (2.40–3.59) | <0.001 | 2.88 (2.35–3.52) | <0.001 | 2.64 (2.14–3.26) | <0.001 | 120 |
| Elevated FPG | 1.11 (0.88–1.40) | 0.38 | 1.09 (0.86–1.39) | 0.45 | 1.08 (0.84–1.39) | 0.56 | 60 |
| Elevated TG | 1.55 (1.33–1.80) | <0.001 | 1.65 (1.40–1.95) | <0.001 | 1.43 (1.20–1.71) | <0.001 | 162 |
| HDL-C<1.03 or non-HDL-C≥3.76 mmol/L (CN2025) | 1.60 (1.39–1.86) | <0.001 | 1.63 (1.40–1.89) | <0.001 | 1.65 (1.41–1.94) | <0.001 | 553 |
| HDL-C<1.03 mmol/L (IDF) | 1.46 (1.28–1.66) | <0.001 | 1.47 (1.28–1.68) | <0.001 | 1.48 (1.28–1.72) | <0.001 | 414 |
| Hypertension (CN2025) | 1.33 (1.17–1.51) | <0.001 | 1.33 (1.17–1.51) | <0.001 | 1.27 (1.11–1.46) | <0.001 | 367 |
| Hypertension (IDF) | 1.42 (1.24–1.63) | <0.001 | 1.39 (1.21–1.59) | <0.001 | 1.31 (1.13–1.51) | <0.001 | 243 |

Data are presented as OR (95% CI). Model 1: unadjusted. Model 2: adjusted for age and sex. Model 3: adjusted for age, sex, ALT, AST, and family history of T2DM. All P values < 0.001 except for elevated FPG.

**Table S2. Comparison of AUCs between BRI and BMI for predicting MetS and its metabolic components.**

| **Outcome** | **BRI AUC** | **BMI** **AUC** | **Δ AUC** | **P_value** | **Events** |
| --- | --- | --- | --- | --- | --- |
| MetS (CN2025) | 0.8729 | 0.8352 | 0.0377 | 0.005 | 193 |
| MetS (IDF) | 0.8586 | 0.8455 | 0.013 | 0.309 | 120 |
| Elevated FPG | 0.7219 | 0.7221 | -0.0002 | 0.933 | 60 |
| Elevated TG | 0.7908 | 0.7907 | 0.0001 | 0.984 | 162 |
| Hypertension (CN2025) | 0.5787 | 0.6321 | -0.0534 | <0.001 | 367 |
| Hypertension (IDF) | 0.6403 | 0.6725 | -0.0322 | 0.002 | 243 |
| Low HDL-C or Elevated non-HDL-C (CN2025) | 0.6785 | 0.6554 | 0.0231 | 0.01263 | 553 |
| Low HDL (IDF) | 0.6697 | 0.6548 | 0.0149 | 0.040 | 414 |

AUCs were obtained from Model 3 (adjusted for age, sex, ALT, AST, and family history of T2DM). ΔAUC = BRI AUC − BMI AUC. P values for AUC comparison were derived from the DeLong test.

**Table S3. Distribution of serum biomarkers across BRI quartiles.**

|  | **Q1** | **Q2** | **Q3** | **Q4** | **P** |
| --- | --- | --- | --- | --- | --- |
| AFABP (μg/L) | 2.74 (2.32, 3.64) | 2.96 (2.32, 3.69) | 2.90 (2.31, 3.56) | 2.85 (2.26, 3.76) | 0.74 |
| ApoB48 (μg/L) | 49.51 (36.98, 77.25) | 55.91 (36.94, 103.92) | 49.55 (31.27, 83.09) | 45.14 (23.02, 79.60) | 0.01 |
| RBP4 (mg/L) | 1.20 (1.05, 1.54) | 1.24 (1.03, 1.71) | 1.20 (0.99, 1.53) | 1.24 (1.05, 1.58) | 0.31 |
| Resistin (μg/L) | 27.58 (14.83, 47.34) | 28.84 (15.47, 51.91) | 26.23 (13.19, 43.21) | 31.30 (14.85, 56.90) | 0.31 |
| NPY (ng/L) | 1242.99±821.26 | 1255.73±768.27 | 1123.79±744.59 | 1238.16±688.95 | 0.28 |
| PAI-1 (ng/mL) | 18.88 (9.40, 33.59) | 22.86 (10.61, 38.71) | 20.37 (8.73, 35.84) | 24.13 (10.20, 34.02) | 0.39 |
| AgRP (pg/mL) | 1838.62 (1137.58, 2661.20) | 2010.97 (1024.22, 2964.95) | 1896.91 (905.47, 2955.04) | 1908.62 (1141.70, 2889.07) | 0.7 |
| CTRP9 (pg/mL) | 101.07 (66.48, 214.99) | 119.06 (69.95, 218.46) | 93.38 (59.98, 184.13) | 100.22 (65.47, 198.88) | 0.26 |
| Irisin (ng/mL) | 44.42 (21.41, 74.25) | 46.78 (23.65, 74.10) | 40.36 (20.45, 59.55) | 39.65 (20.37, 60.13) | 0.1 |
| Pentraxin-3 (ng/mL) | 13.94 (7.36, 21.67) | 15.60 (9.93, 22.10) | 14.14 (8.18, 20.11) | 13.52 (6.87, 18.54) | 0.14 |
| SFRP5 (pg/mL) | 83.83 (48.55, 171.51) | 132.94 (73.66, 237.71) | 106.05 (63.11, 174.12) | 99.29 (49.13, 171.59) | 0.006 |
| TNF-α (pg/mL) | 517.84 (252.68, 861.87) | 476.23 (252.71, 819.90) | 474.34 (195.55, 866.52) | 503.90 (231.29, 974.12) | 0.8 |
| Lipocalin-2 (μg/L) | 8.54 (4.64, 13.71) | 9.28 (5.01, 14.86) | 8.83 (3.88, 15.35) | 9.92 (4.68, 16.33) | 0.48 |
| hs-CRP (μg/mL) | 4.87±3.96 | 5.16±4.21 | 4.26±3.63 | 2.84 (1.51, 5.42) | 0.04 |
| Visfatin (μg/L) | 25.09±15.13 | 24.40 (13.09, 35.67) | 26.57±17.27 | 24.74±14.19 | 0.91 |
| Leptin (μg/L) | 17.16±16.18 | 17.20±16.11 | 17.36±16.56 | 20.89±16.77 | 0.12 |
| Obestatin (ng/L) | 564.77±310.45 | 462.39 (287.35, 700.69) | 495.32 (276.73, 719.76) | 529.25±304.51 | 0.58 |
| Homocysteine (μmol/L) | 22.80±18.10 | 25.01±18.97 | 21.86±17.39 | 19.76±15.79 | 0.11 |
| UA (μmol/L) | 291.77±82.82 | 296.83±79.23 | 304.88±87.88 | 354.87±98.79 | <0.001 |
| FINS (mIU/L) | 15.00 (11.00, 20.50) | 17.00 (11.50, 23.00) | 18.50 (14.50, 24.00) | 22.00 (16.88, 30.00) | <0.001 |
| AST (U/L) | 16.00 (15.00, 18.00) | 17.00 (14.00, 19.00) | 17.00 (14.00, 19.00) | 17.00 (15.00, 20.00) | 0.03 |
| ALT (U/L) | 10.00 (8.00, 12.00) | 10.00 (7.95, 12.00) | 10.00 (8.00, 13.00) | 13.00 (10.00, 20.00) | <0.001 |

Data are presented as median (interquartile range) for non-normally distributed variables or mean ± standard deviation for normally distributed variables. P values are from Kruskal–Wallis tests or one-way ANOVA, as appropriate.

**Table S4. Parallel mediation analysis of serum biomarker modules in the BRI-MetS component relationship.**

| Outcome | Mediator | IE | PM (%) | p-value |  | Total OR | Direct OR | Combined IE | Overall PM(%) |
| --- | --- | --- | --- | --- | --- | --- | --- | --- | --- |
| *Upward module* | | | | | | | | | |
| Hypertension (CN2025) |  |  |  |  |  | 1.245 | 1.108 | 0.1184 | 54.1% |
|  | Leptin | +0.0157[-0.0131,+0.0524] | 7.2% | 0.2720 |  |  |  |  |  |
|  | UA | +0.0384[-0.0052,+0.0921] | 17.5% | 0.0790 |  |  |  |  |  |
|  | FINS | +0.0581[-0.0074,+0.1517] | 26.5% | 0.0890 |  |  |  |  |  |
|  | RBP4 | +0.0001[-0.0148,+0.0207] | 0.0% | 0.9820 |  |  |  |  |  |
|  | Resistin | +0.0057[-0.0110,+0.0302] | 2.6% | 0.4990 |  |  |  |  |  |
|  | NPY | +0.0012[-0.0182,+0.0205] | 0.5% | 0.9130 |  |  |  |  |  |
|  | AgRP | -0.0008[-0.0105,+0.0076] | -0.4% | 0.8740 |  |  |  |  |  |
| Hypertension (IDF) |  |  |  |  |  | 1.283 | 1.089 | 0.1480 | 59.5% |
|  | Leptin | +0.0121[-0.0227,+0.0534] | 4.9% | 0.4690 |  |  |  |  |  |
|  | UA | +0.0264[-0.0183,+0.0752] | 10.6% | 0.2520 |  |  |  |  |  |
|  | FINS | +0.0773[+0.0037,+0.1883] | 31.1% | 0.0370 | * |  |  |  |  |
|  | AST | -0.0134[-0.0788,+0.0476] | -5.4% | 0.5400 |  |  |  |  |  |
|  | ALT | +0.0476[-0.0749,+0.2037] | 19.1% | 0.4540 |  |  |  |  |  |
|  | RBP4 | +0.0001[-0.0221,+0.0139] | 0.0% | 0.9060 |  |  |  |  |  |
|  | Resistin | +0.0141[-0.0071,+0.0556] | 5.7% | 0.2400 |  |  |  |  |  |
|  | NPY | +0.0007[-0.0143,+0.0175] | 0.3% | 0.9280 |  |  |  |  |  |
|  | PAI-1 | +0.0053[-0.0085,+0.0255] | 2.1% | 0.4700 |  |  |  |  |  |
|  | AgRP | -0.0021[-0.0189,+0.0105] | -0.8% | 0.7630 |  |  |  |  |  |
|  | TNF-α | -0.0086[-0.0468,+0.0170] | -3.4% | 0.4990 |  |  |  |  |  |
|  | Lipo-2 | -0.0116[-0.0470,+0.0062] | -4.6% | 0.2910 |  |  |  |  |  |
| Abn HDL/non-HDL (CN2025) |  |  |  |  |  | 1.691 | 1.712 | 0.0050 | 0.9% |
|  | Leptin | -0.0374[-0.0862,-0.0047] | -7.1% | 0.0190 | * |  |  |  |  |
|  | UA | -0.0024[-0.0459,+0.0422] | -0.5% | 0.9080 |  |  |  |  |  |
|  | FINS | +0.0067[-0.0739,+0.0920] | 1.3% | 0.7900 |  |  |  |  |  |
|  | AST | +0.0102[-0.0311,+0.0590] | 1.9% | 0.6600 |  |  |  |  |  |
|  | RBP4 | +0.0000[-0.0156,+0.0421] | 0.0% | 0.7400 |  |  |  |  |  |
|  | Resistin | +0.0127[-0.0061,+0.0507] | 2.4% | 0.2270 |  |  |  |  |  |
|  | NPY | +0.0006[-0.0124,+0.0147] | 0.1% | 0.9610 |  |  |  |  |  |
|  | PAI-1 | +0.0222[+0.0010,+0.0642] | 4.2% | 0.0380 | * |  |  |  |  |
|  | AgRP | -0.0029[-0.0207,+0.0141] | -0.6% | 0.7060 |  |  |  |  |  |
|  | TNF-α | -0.0088[-0.0440,+0.0172] | -1.7% | 0.4750 |  |  |  |  |  |
|  | Lipo-2 | +0.0040[-0.0139,+0.0240] | 0.8% | 0.6930 |  |  |  |  |  |
| Low HDL (IDF) |  |  |  |  |  | 1.372 | 1.337 | 0.0220 | 7.0% |
|  | FINS | +0.0225[-0.0414,+0.0863] | 7.1% | 0.4720 |  |  |  |  |  |
|  | NPY | -0.0027[-0.0222,+0.0127] | -0.9% | 0.7200 |  |  |  |  |  |
|  | PAI-1 | +0.0022[-0.0104,+0.0244] | 0.7% | 0.6580 |  |  |  |  |  |
| Elev FPG |  |  |  |  |  | 1.092 | 0.960 | 0.0966 | 109.2% |
|  | FINS | +0.0966[-0.0040,+0.1846] | 109.2% | 0.0550 |  |  |  |  |  |
| Elev TG |  |  |  |  |  | 1.650 | 1.281 | 0.2649 | 52.9% |
|  | UA | +0.0637[+0.0199,+0.1160] | 12.7% | 0.0050 | * |  |  |  |  |
|  | FINS | +0.0696[+0.0091,+0.1388] | 13.9% | 0.0210 | * |  |  |  |  |
|  | ALT | +0.1317[+0.0678,+0.2396] | 26.3% | 0.0000 | * |  |  |  |  |
| *Declining module* | | | | | | | | | |
| Hypertension (CN2025) |  |  |  |  |  | 1.346 | 1.328 | 0.0249 | 8.4% |
|  | ApoB48 | +0.0120[-0.0082,+0.0430] | 4.0% | 0.2620 |  |  |  |  |  |
|  | Irisin | +0.0129[-0.0071,+0.0406] | 4.3% | 0.2230 |  |  |  |  |  |
| Hypertension (IDF) |  |  |  |  |  | 1.395 | 1.395 | -0.0019 | -0.6% |
|  | AFABP | -0.0057[-0.0289,+0.0091] | -1.7% | 0.4670 |  |  |  |  |  |
|  | ApoB48 | +0.0064[-0.0090,+0.0295] | 1.9% | 0.4160 |  |  |  |  |  |
|  | CTRP9 | +0.0080[-0.0117,+0.0422] | 2.4% | 0.4320 |  |  |  |  |  |
|  | Irisin | +0.0171[-0.0166,+0.0701] | 5.1% | 0.3330 |  |  |  |  |  |
|  | PTX-3 | -0.0181[-0.0606,+0.0153] | -5.4% | 0.2460 |  |  |  |  |  |
|  | SFRP5 | +0.0000[-0.0361,+0.0313] | 0.0% | 0.9960 |  |  |  |  |  |
|  | Hcy | -0.0097[-0.0387,+0.0090] | -2.9% | 0.3530 |  |  |  |  |  |
| Abn HDL/non-HDL (CN2025) |  |  |  |  |  | 1.647 | 1.600 | -0.0179 | -3.6% |
|  | AFABP | -0.0037[-0.0231,+0.0096] | -0.7% | 0.5950 |  |  |  |  |  |
|  | ApoB48 | -0.0060[-0.0280,+0.0060] | -1.2% | 0.3620 |  |  |  |  |  |
|  | Irisin | +0.0411[+0.0101,+0.0936] | 8.2% | 0.0070 | * |  |  |  |  |
|  | PTX-3 | -0.0748[-0.1547,-0.0285] | -15.0% | 0.0000 | * |  |  |  |  |
|  | hs-CRP | +0.0052[-0.0188,+0.0360] | 1.0% | 0.6770 |  |  |  |  |  |
|  | Hcy | +0.0204[-0.0018,+0.0539] | 4.1% | 0.0770 |  |  |  |  |  |
| Low HDL (IDF) |  |  |  |  |  | 1.333 | 1.313 | -0.0122 | -4.3% |
|  | AFABP | -0.0022[-0.0181,+0.0115] | -0.7% | 0.7060 |  |  |  |  |  |
|  | ApoB48 | -0.0095[-0.0368,+0.0109] | -3.3% | 0.3420 |  |  |  |  |  |
|  | Irisin | +0.0022[-0.0290,+0.0410] | 0.7% | 0.8570 |  |  |  |  |  |
|  | PTX-3 | -0.0284[-0.0757,+0.0046] | -9.9% | 0.0910 |  |  |  |  |  |
|  | SFRP5 | -0.0136[-0.0525,+0.0120] | -4.7% | 0.3090 |  |  |  |  |  |
|  | hs-CRP | +0.0031[-0.0268,+0.0337] | 1.1% | 0.8490 |  |  |  |  |  |
|  | Hcy | +0.0361[+0.0013,+0.0928] | 12.6% | 0.0470 | * |  |  |  |  |
| Elev FPG |  |  |  |  |  | 1.264 | 1.308 | 0.1187 | 50.7% |
|  | AFABP | -0.0030[-0.0367,+0.0294] | -1.3% | 0.8640 |  |  |  |  |  |
|  | ApoB48 | +0.0120[-0.0184,+0.0475] | 5.1% | 0.3880 |  |  |  |  |  |
|  | CTRP9 | -0.0153[-0.0653,+0.0187] | -6.5% | 0.4080 |  |  |  |  |  |
|  | Irisin | +0.0075[-0.0489,+0.0819] | 3.2% | 0.7870 |  |  |  |  |  |
|  | PTX-3 | +0.0537[-0.0518,+0.3092] | 22.9% | 0.3870 |  |  |  |  |  |
|  | SFRP5 | +0.1084[+0.0265,+0.2776] | 46.3% | 0.0050 | * |  |  |  |  |
|  | hs-CRP | -0.0591[-0.1435,-0.0037] | -25.2% | 0.0320 | * |  |  |  |  |
|  | Hcy | +0.0144[-0.0205,+0.0693] | 6.2% | 0.4270 |  |  |  |  |  |
| Elev TG |  |  |  |  |  | 1.556 | 1.530 | 0.0725 | 16.4% |
|  | PTX-3 | +0.0301[-0.0140,+0.1156] | 6.8% | 0.1770 |  |  |  |  |  |
|  | SFRP5 | +0.0118[-0.0205,+0.0552] | 2.7% | 0.4850 |  |  |  |  |  |
|  | hs-CRP | +0.0307[+0.0023,+0.0779] | 6.9% | 0.0330 | * |  |  |  |  |

he Upward module (leptin, UA, FINS, AST, ALT, RBP4, resistin, NPY, PAI-1, AgRP, TNF-α, lipocalin-2) and Declining module (AFABP, ApoB48, CTRP9, irisin, pentraxin-3, SFRP5, hs-CRP, homocysteine) were tested as parallel mediators between BRI (exposure) and each MetS component (outcome). Mediators within each module were selected via LASSO regression. All models were adjusted for age, sex, and family history of type 2 diabetes. Total effect (Total OR), direct effect (Direct OR), indirect effect (IE), combined indirect effect (Combined IE), and proportion mediated [PM (%) = Combined IE / ln(Total OR)] are reported. For individual mediators, bootstrap 95% confidence intervals and p-values are shown. *P < 0.05.

**Table S5. Characteristics of participants with and without serum biomarker data**

| **Variable** | **With biomarker data (n=683)** | **Without biomarker data (n=231)** | **P** |
| --- | --- | --- | --- |
| Age, years | 13.0 (12.0–15.0) | 14.0 (13.0–15.0) | <0.001 |
| Sex, male | 368 (53.9%) | 118 (51.1%) | 0.509 |
| BMI, kg/m² | 20.5 (18.6–23.6) | 21.0 (18.5–24.9) | 0.261 |
| BRI | 2.6 (2.1–3.4) | 2.9 (2.1–3.5) | 0.066 |
| WC, cm | 74.5 (69.5–81.5) | 77.5 (69.8–85.5) | 0.016 |
| MetS (CN2025) | 136 (19.9%) | 57 (24.7%) | 0.150 |
| MetS (IDF) | 80 (11.7%) | 40 (17.3%) | 0.039 |

Continuous variables are median (IQR), compared by Mann–Whitney U test; categorical variables are n (%), compared by chi-square test. Abbreviations: BMI, body mass index; BRI, body roundness index; WC, waist circumference; MetS, metabolic syndrome; CN2025, Chinese pediatric criteria; IDF, International Diabetes Federation.
